# Supplementary material for: Modeling glioblastoma heterogeneity as a dynamic network of cell states
Source: Mol Syst Biol. 2021 Sep 16;17(9):e10105. doi: 10.15252/msb.202010105 (PMC8444284; doi:10.15252/msb.202010105)
Supplement: Supplementary file 5 — Source Data for Figure 3 [file MSB-17-e10105-s001.zip › Figure3A_sourcedata/GSEA_3065/nosigdb_state5.GseaPreranked.1622718435914/gsea_report_for_na_neg_1622718435914.html]

Report for na\_neg 1622718435914 [GSEA]

| GS  follow link to MSigDB | GS DETAILS | SIZE | ES | NES | NOM p-val | FDR q-val | FWER p-val | RANK AT MAX | LEADING EDGE || 1 | LARSSON\_STATE\_1 | Details ... | 99 | -0.80 | -2.76 | 0.000 | 0.000 | 0.000 | 967 | tags=73%, list=11%, signal=81% |
| 2 | NB\_KI\_CLUSTER1 | Details ... | 32 | -0.96 | -2.71 | 0.000 | 0.000 | 0.000 | 272 | tags=97%, list=3%, signal=100% |
| 3 | LARSSON\_STATE\_6 | Details ... | 101 | -0.76 | -2.65 | 0.000 | 0.000 | 0.000 | 1165 | tags=71%, list=13%, signal=81% |
| 4 | OLSEN\_ADRENERGIC | Details ... | 65 | -0.76 | -2.44 | 0.000 | 0.000 | 0.000 | 1121 | tags=69%, list=12%, signal=79% |
| 5 | RICHARDS\_NOWAKOWSKI\_SCIENCE\_2017\_RG\_EARLY\_UPREG | Details ... | 432 | -0.61 | -2.41 | 0.000 | 0.000 | 0.000 | 1134 | tags=37%, list=13%, signal=41% |
| 6 | LARSSON\_STATE\_4 | Details ... | 101 | -0.69 | -2.39 | 0.000 | 0.000 | 0.000 | 1458 | tags=51%, list=16%, signal=61% |
| 7 | RICHARDS\_NOWAKOWSKI\_SCIENCE\_2017\_MGE\_RG2\_UPREG | Details ... | 436 | -0.57 | -2.22 | 0.000 | 0.000 | 0.000 | 1014 | tags=32%, list=11%, signal=34% |
| 8 | NB\_KI\_CLUSTER3 | Details ... | 161 | -0.58 | -2.10 | 0.000 | 0.000 | 0.000 | 1719 | tags=46%, list=19%, signal=56% |
| 9 | DOROSZKO\_U3180MG\_CLUSTER\_3 | Details ... | 468 | -0.52 | -2.05 | 0.000 | 0.000 | 0.000 | 1799 | tags=41%, list=20%, signal=48% |
| 10 | NB\_KI\_CLUSTER4 | Details ... | 90 | -0.60 | -2.04 | 0.000 | 0.000 | 0.000 | 1121 | tags=48%, list=12%, signal=54% |
| 11 | FELDMAN\_2020\_S-G2 | Details ... | 328 | -0.53 | -2.04 | 0.000 | 0.000 | 0.000 | 1458 | tags=44%, list=16%, signal=50% |
| 12 | RICHARDS\_NOWAKOWSKI\_SCIENCE\_2017\_IPC\_NEN3\_UPREG | Details ... | 229 | -0.53 | -2.01 | 0.000 | 0.000 | 0.000 | 1238 | tags=33%, list=14%, signal=37% |
| 13 | DOROSZKO\_U3180MG\_CLUSTER\_4 | Details ... | 166 | -0.54 | -1.95 | 0.000 | 0.000 | 0.005 | 1764 | tags=45%, list=20%, signal=54% |
| 14 | FELDMAN\_2020\_G2-M | Details ... | 286 | -0.50 | -1.89 | 0.000 | 0.001 | 0.011 | 1148 | tags=34%, list=13%, signal=38% |
| 15 | RICHARDS\_NOWAKOWSKI\_SCIENCE\_2017\_MGE\_IPC3\_UPREG | Details ... | 115 | -0.53 | -1.86 | 0.000 | 0.001 | 0.019 | 694 | tags=28%, list=8%, signal=30% |
| 16 | FELDMAN\_2020\_NEURAL G0 | Details ... | 370 | -0.48 | -1.86 | 0.000 | 0.001 | 0.021 | 1163 | tags=38%, list=13%, signal=42% |
| 17 | NB\_KI\_CLUSTER5 | Details ... | 86 | -0.53 | -1.81 | 0.000 | 0.002 | 0.044 | 1434 | tags=37%, list=16%, signal=44% |
| 18 | FELDMAN\_2020\_S | Details ... | 151 | -0.49 | -1.79 | 0.000 | 0.002 | 0.061 | 2060 | tags=50%, list=23%, signal=63% |
| 19 | FELDMAN\_2020\_G1 | Details ... | 149 | -0.48 | -1.75 | 0.000 | 0.003 | 0.093 | 1201 | tags=34%, list=13%, signal=39% |
| 20 | FELDMAN\_2020\_M-EARLYG1 | Details ... | 108 | -0.50 | -1.74 | 0.001 | 0.004 | 0.119 | 1122 | tags=47%, list=12%, signal=53% |
| 21 | DOROSZKO\_U3013MG\_CLUSTER\_2 |  | 206 | -0.46 | -1.73 | 0.000 | 0.005 | 0.136 | 2618 | tags=46%, list=29%, signal=63% |
| 22 | RICHARDS\_NOWAKOWSKI\_SCIENCE\_2017\_IPC\_NEN1\_UPREG\_1 |  | 133 | -0.47 | -1.69 | 0.001 | 0.007 | 0.222 | 1136 | tags=26%, list=13%, signal=30% |
| 23 | RICHARDS\_ZHONG\_NATURE\_2018\_NPCS\_UPREG |  | 124 | -0.48 | -1.69 | 0.000 | 0.007 | 0.222 | 1458 | tags=30%, list=16%, signal=35% |
| 24 | RICHARDS\_VERHAAK\_CANCERCELL\_2010\_NEURAL |  | 60 | -0.52 | -1.66 | 0.000 | 0.010 | 0.301 | 2178 | tags=38%, list=24%, signal=50% |
| 25 | RICHARDS\_NEFTEL\_CELL\_2019\_MES1 |  | 38 | -0.55 | -1.65 | 0.011 | 0.011 | 0.330 | 618 | tags=37%, list=7%, signal=39% |
| 26 | SUVA\_MES1\_PMID\_31327527 |  | 38 | -0.55 | -1.62 | 0.006 | 0.014 | 0.425 | 618 | tags=37%, list=7%, signal=39% |
| 27 | NB\_KI\_CLUSTER11 |  | 50 | -0.53 | -1.61 | 0.000 | 0.014 | 0.451 | 1119 | tags=34%, list=12%, signal=39% |
| 28 | OLSEN\_MESENCHYMAL |  | 70 | -0.48 | -1.60 | 0.001 | 0.015 | 0.483 | 531 | tags=27%, list=6%, signal=29% |
| 29 | RICHARDS\_NOWAKOWSKI\_SCIENCE\_2017\_NIN4\_UPREG |  | 132 | -0.45 | -1.60 | 0.001 | 0.015 | 0.487 | 1032 | tags=25%, list=11%, signal=28% |
| 30 | GAROFANO\_IAVARONE\_LIST\_OF\_NEURAL\_PROGENITOR\_MARKER\_GENES |  | 83 | -0.47 | -1.59 | 0.001 | 0.016 | 0.505 | 1976 | tags=42%, list=22%, signal=54% |
| 31 | RICHARDS\_NOWAKOWSKI\_SCIENCE\_2017\_IN\_CTX\_CGE1\_UPREG |  | 294 | -0.40 | -1.52 | 0.001 | 0.031 | 0.769 | 1539 | tags=27%, list=17%, signal=32% |
| 32 | SUVA\_G1S\_PMID\_31327527 |  | 27 | -0.54 | -1.50 | 0.026 | 0.038 | 0.851 | 2688 | tags=52%, list=30%, signal=74% |
| 33 | RICHARDS\_NEFTEL\_CELL\_2019\_G1\_S |  | 27 | -0.54 | -1.49 | 0.037 | 0.041 | 0.877 | 2688 | tags=52%, list=30%, signal=74% |
| 34 | FELDMAN\_2020\_LATEG1 |  | 91 | -0.44 | -1.48 | 0.009 | 0.043 | 0.892 | 1134 | tags=30%, list=13%, signal=34% |
| 35 | IVY\_IT |  | 33 | -0.50 | -1.45 | 0.054 | 0.052 | 0.939 | 2062 | tags=42%, list=23%, signal=55% |
| 36 | NB\_KI\_CLUSTER16 |  | 72 | -0.44 | -1.44 | 0.028 | 0.055 | 0.948 | 1466 | tags=28%, list=16%, signal=33% |
| 37 | DALMO\_BARCODENUMBER\_UNPUB |  | 190 | -0.38 | -1.39 | 0.007 | 0.081 | 0.991 | 1496 | tags=32%, list=17%, signal=38% |
| 38 | RICHARDS\_NEFTEL\_CELL\_2019\_NPC2 |  | 32 | -0.48 | -1.38 | 0.068 | 0.084 | 0.994 | 1320 | tags=31%, list=15%, signal=37% |
| 39 | RICHARDS\_NEFTEL\_CELL\_2019\_MES2 |  | 44 | -0.46 | -1.38 | 0.065 | 0.082 | 0.995 | 1036 | tags=27%, list=12%, signal=31% |
| 40 | RICHARDS\_NOWAKOWSKI\_SCIENCE\_2017\_NEN\_LATE\_UPREG |  | 355 | -0.36 | -1.38 | 0.003 | 0.081 | 0.995 | 1154 | tags=22%, list=13%, signal=24% |
| 41 | SUVA\_MES2\_PMID\_31327527 |  | 44 | -0.46 | -1.38 | 0.070 | 0.084 | 0.995 | 1036 | tags=27%, list=12%, signal=31% |
| 42 | RICHARDS\_NOWAKOWSKI\_SCIENCE\_2017\_IN\_CTX\_MGE1\_UPREG |  | 276 | -0.36 | -1.36 | 0.010 | 0.092 | 0.999 | 1631 | tags=27%, list=18%, signal=32% |
| 43 | SUVA\_NPC2\_PMID\_31327527 |  | 32 | -0.48 | -1.36 | 0.103 | 0.091 | 0.999 | 1320 | tags=31%, list=15%, signal=37% |
| 44 | DOROSZKO\_U3013MG\_CLUSTER\_3 |  | 99 | -0.38 | -1.32 | 0.058 | 0.118 | 1.000 | 1564 | tags=46%, list=17%, signal=56% |
| 45 | DOROSZKO\_U3054MG\_CLUSTER\_6 |  | 113 | -0.38 | -1.32 | 0.057 | 0.116 | 1.000 | 2256 | tags=39%, list=25%, signal=51% |
| 46 | RICHARDS\_LIDDELOW\_NATURE\_2017\_A2\_REACTIVE\_ASTROCYTES |  | 29 | -0.46 | -1.29 | 0.139 | 0.145 | 1.000 | 436 | tags=24%, list=5%, signal=25% |
| 47 | VANGRONINGEN\_ADRN |  | 230 | -0.33 | -1.26 | 0.046 | 0.178 | 1.000 | 2080 | tags=32%, list=23%, signal=40% |
| 48 | RICHARDS\_NOWAKOWSKI\_SCIENCE\_2017\_IN\_CTX\_MGE2\_UPREG |  | 193 | -0.34 | -1.26 | 0.068 | 0.174 | 1.000 | 1228 | tags=23%, list=14%, signal=26% |
| 49 | DOROSZKO\_U3180MG\_CLUSTER\_5 |  | 31 | -0.44 | -1.24 | 0.181 | 0.204 | 1.000 | 1873 | tags=52%, list=21%, signal=65% |
| 50 | RICHARDS\_NOWAKOWSKI\_SCIENCE\_2017\_EN\_PFC3\_UPREG |  | 198 | -0.33 | -1.24 | 0.083 | 0.202 | 1.000 | 1740 | tags=28%, list=19%, signal=34% |
| 51 | GAROFANO\_IAVARONE\_MTC\_SUBTYPE |  | 216 | -0.32 | -1.22 | 0.089 | 0.219 | 1.000 | 927 | tags=14%, list=10%, signal=15% |
| 52 | SEURAT\_CELLCYCLE |  | 89 | -0.35 | -1.20 | 0.162 | 0.254 | 1.000 | 2775 | tags=39%, list=31%, signal=56% |
| 53 | WENG\_STEMNESS\_PMID\_30982771 |  | 36 | -0.40 | -1.18 | 0.234 | 0.276 | 1.000 | 388 | tags=17%, list=4%, signal=17% |
| 54 | GANDAL\_ALCOHOLADDICTION\_UP\_PMID29439242 |  | 136 | -0.33 | -1.18 | 0.151 | 0.278 | 1.000 | 1199 | tags=20%, list=13%, signal=23% |
| 55 | RICHARDS\_NOWAKOWSKI\_SCIENCE\_2017\_MGE\_IPC2\_UPREG |  | 479 | -0.29 | -1.16 | 0.106 | 0.308 | 1.000 | 1458 | tags=19%, list=16%, signal=21% |
| 56 | SINGH\_SOX2\_DOWNREGULATED\_TRANSCRIPTS\_PMID\_28122245 |  | 88 | -0.33 | -1.13 | 0.247 | 0.361 | 1.000 | 1932 | tags=27%, list=22%, signal=34% |
| 57 | RICHARDS\_NEFTEL\_CELL\_2019\_G2\_M |  | 43 | -0.36 | -1.11 | 0.313 | 0.398 | 1.000 | 3382 | tags=56%, list=38%, signal=89% |
| 58 | RICHARDS\_NOWAKOWSKI\_SCIENCE\_2017\_NIN5\_UPREG |  | 256 | -0.29 | -1.10 | 0.241 | 0.408 | 1.000 | 795 | tags=14%, list=9%, signal=15% |
| 59 | SUVA\_G2M\_PMID\_31327527 |  | 43 | -0.36 | -1.08 | 0.349 | 0.445 | 1.000 | 3382 | tags=56%, list=38%, signal=89% |
| 60 | IVY\_LE |  | 27 | -0.39 | -1.08 | 0.355 | 0.452 | 1.000 | 1128 | tags=26%, list=13%, signal=30% |
| 61 | GANDAL\_AUTISM\_UP\_PMID29439242 |  | 81 | -0.32 | -1.07 | 0.369 | 0.470 | 1.000 | 1590 | tags=27%, list=18%, signal=33% |
| 62 | WANG\_PRONEURAL\_PMID\_28697342 |  | 15 | -0.44 | -1.06 | 0.385 | 0.468 | 1.000 | 1121 | tags=33%, list=12%, signal=38% |
| 63 | GAROFANO\_IAVARONE\_PPR\_SUBTYPE |  | 396 | -0.27 | -1.06 | 0.315 | 0.466 | 1.000 | 1458 | tags=16%, list=16%, signal=18% |
| 64 | NB\_KI\_CLUSTER18 |  | 70 | -0.32 | -1.05 | 0.374 | 0.488 | 1.000 | 577 | tags=19%, list=6%, signal=20% |
| 65 | SUVA\_NPC1\_PMID\_31327527 |  | 34 | -0.35 | -1.03 | 0.406 | 0.520 | 1.000 | 441 | tags=18%, list=5%, signal=18% |
| 66 | RICHARDS\_VERHAAK\_CANCERCELL\_2010\_MESENCHYMAL |  | 99 | -0.30 | -1.03 | 0.424 | 0.525 | 1.000 | 1463 | tags=20%, list=16%, signal=24% |
| 67 | RICHARDS\_NEFTEL\_CELL\_2019\_NPC1 |  | 34 | -0.35 | -1.02 | 0.457 | 0.542 | 1.000 | 441 | tags=18%, list=5%, signal=18% |
| 68 | RICHARDS\_NOWAKOWSKI\_SCIENCE\_2017\_NIN3\_UPREG |  | 138 | -0.27 | -0.98 | 0.519 | 0.626 | 1.000 | 1330 | tags=22%, list=15%, signal=25% |
| 69 | ALMSTEDT\_11Q\_MSIGDB |  | 302 | -0.25 | -0.96 | 0.556 | 0.662 | 1.000 | 1260 | tags=17%, list=14%, signal=19% |
| 70 | DALMO\_EXTINCTION\_UNPUB |  | 24 | -0.35 | -0.96 | 0.524 | 0.673 | 1.000 | 1411 | tags=21%, list=16%, signal=25% |
| 71 | WANG\_MESENCHYMAL\_PMID\_28697342 |  | 24 | -0.35 | -0.96 | 0.517 | 0.665 | 1.000 | 1411 | tags=21%, list=16%, signal=25% |
| 72 | RICHARDS\_NOWAKOWSKI\_SCIENCE\_2017\_NIN1\_UPREG |  | 147 | -0.26 | -0.94 | 0.587 | 0.683 | 1.000 | 457 | tags=11%, list=5%, signal=11% |
| 73 | ALMSTEDT\_17Q\_MSIGDB |  | 350 | -0.23 | -0.90 | 0.722 | 0.764 | 1.000 | 2538 | tags=32%, list=28%, signal=42% |
| 74 | PARADA\_T1\_UP\_26461091 |  | 62 | -0.28 | -0.90 | 0.617 | 0.759 | 1.000 | 1069 | tags=21%, list=12%, signal=24% |
| 75 | PARADA\_T2\_UP\_26461091 |  | 62 | -0.28 | -0.90 | 0.667 | 0.766 | 1.000 | 680 | tags=11%, list=8%, signal=12% |
| 76 | KLING\_GBM\_PHENO\_PMID\_27667176 |  | 142 | -0.24 | -0.87 | 0.726 | 0.800 | 1.000 | 1168 | tags=15%, list=13%, signal=17% |
| 77 | DOROSZKO\_U3180MG\_CLUSTER\_6 |  | 39 | -0.29 | -0.86 | 0.693 | 0.817 | 1.000 | 2324 | tags=41%, list=26%, signal=55% |
| 78 | IVY\_CTPAN |  | 101 | -0.25 | -0.86 | 0.748 | 0.809 | 1.000 | 1087 | tags=17%, list=12%, signal=19% |
| 79 | GANDAL\_IBD\_DN\_PMID29439242 |  | 19 | -0.32 | -0.81 | 0.746 | 0.886 | 1.000 | 1906 | tags=37%, list=21%, signal=47% |
| 80 | NB\_KI\_CLUSTER13 |  | 112 | -0.22 | -0.77 | 0.898 | 0.935 | 1.000 | 2018 | tags=23%, list=22%, signal=30% |
| 81 | ROSEN\_KRONA\_TI\_UNPUBL |  | 48 | -0.24 | -0.76 | 0.843 | 0.933 | 1.000 | 276 | tags=6%, list=3%, signal=6% |
| 82 | RICHARDS\_CAHOY\_JNEUROSCI\_2011\_ASTRO\_MATURE |  | 483 | -0.18 | -0.73 | 0.998 | 0.949 | 1.000 | 1199 | tags=12%, list=13%, signal=13% |
| 83 | NB\_KI\_CLUSTER12 |  | 39 | -0.25 | -0.72 | 0.891 | 0.953 | 1.000 | 2766 | tags=41%, list=31%, signal=59% |
| 84 | GANDAL\_DEPRESSION\_DN\_PMID29439242 |  | 32 | -0.20 | -0.59 | 0.970 | 0.991 | 1.000 | 1723 | tags=19%, list=19%, signal=23% |
Table: Gene sets enriched in phenotype **na **[plain text format]****
